# Structure and Sensilla of the Mouthparts of the Spotted Lanternfly *Lycorma delicatula* (Hemiptera: Fulgoromorpha: Fulgoridae), a Polyphagous Invasive Planthopper

**DOI:** 10.1371/journal.pone.0156640

**Published:** 2016-06-02

**Authors:** Yanan Hao, Christopher H. Dietrich, Wu Dai

**Affiliations:** 1 Key Laboratory of Plant Protection Resources and Pest Integrated Management of the Ministry of Education, Entomological Museum, Northwest A&F University, Yangling, Shaanxi, China; 2 Illinois Natural History Survey, Prairie Research Institute, University of Illinois at Urbana-Champaign, Illinois, United States of America; Universidad Nacional Autonoma de Mexico, MEXICO

## Abstract

Mouthparts are among the most important sensory and feeding structures in insects and comparative morphological study may help explain differences in feeding behavior and diet breadth among species. The spotted lanternfly *Lycorma delicatula* (White) (Hemiptera: Fulgoromorpha: Fulgoridae) is a polyphagous agricultural pest originating in China, recently established and becoming widespread in Korea, and more recently introduced into eastern North America. It causes severe economic damage by sucking phloem sap and the sugary excrement produced by nymphs and adults serves as a medium for sooty mold. To facilitate future study of feeding mechanisms in this insect, the fine-structural morphology of mouthparts focusing on the distribution of sensilla located on the labium in adult *L*. *delicatula* was observed using a scanning electron microscope. The mouthparts consist of a small cone-shaped labrum, a tubular labium and a stylet fascicle consisting of two inner interlocked maxillary stylets partially surrounded by two shorter mandibular stylets similar to those found in other hemipteran insects. The five-segmented labium is unusual (most other Fulgoromorpha have four segments) and is provided with several types of sensilla and cuticular processes situated on the apex of its distal labial segment. In general, nine types of sensilla were found on the mouthparts. Six types of sensilla and four types of cuticular processes are present on sensory fields of the labial apex. The proposed taxonomic and functional significance of the sensilla are discussed. Morphological similarities in the interlocking mechanism of the stylets suggest a relationship between Fulgoromorpha and Heteroptera.

## Introduction

Hemiptera, a very large and diverse insect order, are united by their specialized piercing-sucking mouthparts that are highly modified for piercing host tissues and extracting their fluid contents. All hemipteran mouthparts comprise the same basic components, a cone-shaped labrum, a tube-like, segmented labium with a deep groove on the anterior side, and a stylet fascicle consisting of two mandibular and two maxillary stylets [1,2]. Abundant data are available on some aspects of mouthpart morphology of Hemiptera based on light and scanning electron microscopy but detailed studies including scanning electron micrographs have been published only for a few species [3–15]. Available data indicate that details of mouthpart morphology, including shape, segmentation and fine structure, vary considerably among hemipteran species and higher taxa and that such differences may be used to distinguish taxa [15–19], provide insight into feeding mechanisms and contribute to assessment of phylogenetic relationships [20]. So far, mouthpart morphology of some major groups remains little studied.

The planthopper superfamily Fulgoroidea (Insecta: Hemiptera) is among the dominant groups of phytophagous hemipterans, comprising >14,000 known species. Many fulgoroid species are economically significant pests of major agricultural crops due to high reproductive potential and capability of transmitting plant pathogens. Previous studies on mouthparts in Fulgoromorpha, which were conducted by scanning electron microscope (SEM) and transmission electron microscope (TEM), have mostly focused upon one aspect such as labial sensilla [1,2,21–25], the interlocking mechanism of maxillae and mandibles [16,17], or gross morphology [10,26], except for a recent study of the delphacid, *Sogatella furcifera* which provided a comprehensive description of the morphology of mouthparts [27]. More detailed and comparative studies of other economically important planthopper species are needed to support research on feeding mechanisms.

The spotted lanternfly, *Lycorma delicatula* (White) (Hemiptera: Auchenorrhyncha: Fulgoridae), was first found in northern China [28–30], and is not only distributed widely throughout China, but has also been reported in Korea [31–34] and was recently detected in the eastern USA [35]. This broad distribution is likely due to its polyphagy and wide range of ecological tolerances, including anthropogenic habitats. Both the nymphs and adults of the spotted lanternfly affect the health of plants primarily through the sucking of phloem sap from the vascular bundles of young stems or leaves [34,36]. In addition, the sugary excretions of this species often result in infestation of the plant by sooty mold, which can interfere with photosynthesis [37]. To facilitate future study of feeding mechanisms and modes of feeding damage, we studied the fine structure of the mouthparts of *L*. *delicatula*.

## Materials and Methods

### Insect Collecting

Adult spotted lanternflies were collected with sweep nets from *Ailanthus altissima* (Mill.) Swingle on the campus of Northwest A&F University in Yangling, Shaanxi Province, China (34°16′N, 108°07′E, elev. 563m) in August 2014, preserved in 70% ethanol and stored at 4°C.

### Scanning Electron Microscopy

Mouthparts of sampled specimens were excised under a stereomicroscope (Olympus SZX10, Japan) using very fine dissecting needles and then dipped into 70% ethanol and cleaned twice using an ultrasonic cleaner (KQ118, Kunshan, China), each time cleaning for one minute and rinsing with 70% ethanol. Samples were then dehydrated in a graded series of 75%, 80%, 85%, 90%, 95% ethanol for 20 min each and in 100% ethanol, twice for 30 min. Specimens were soaked in a graded series of ethanol and tert-Butanol solution, 3:1, 1:1, and 1:3, by volume for 15 min each, and finally in 100% tert-Butanol for 30 min. After removal from the tert-Butanol, the specimens were transferred into a freeze-drier (VFD-21S, SHINKKU VD, Japan) for 3 h. The dried specimens were mounted on aluminum stubs using double-sided copper sticky tape and coated with gold/palladium (40/60) in a high resolution sputter coater (MSP-1S, SHINKKU VD, Japan). The samples were subsequently examined with a Hitachi S-3400N SEM (Hitachi, Tokyo, Japan) operated at 15 kV [38]. Initial observations of 10 females and 10 males of different ages indicated no obvious sex- or age-dependent structural or fine-structural differences other than the tendency for the mouthparts of males to be shorter than those of females, reflecting overall differences in body size. Thus, subsequent observations reported in the results and figures were based on females.

### Image Processing and Morphometric Measurement

Photographs and SEMs were observed and measured after files were imported into Adobe Photoshop CS6. The length of mouthparts was measured from the base of the first labial segment to the end of the fifth segment following Ruttner [39]. The width and height of labial segments were measured from the middle part of each segment. The length of sensilla from base to tip and diameter at the base were measured. Statistical analysis was conducted using SPSS 19.0 (SPSS, Chicago, IL). The terminology for sensilla follows the systems of Altner and Prillinger [40] with more specialized nomenclature for the labial tip sensilla from Brożek and Bourgoin [1].

## Results

### Gross Morphology of Mouthparts

**Fig 1:**
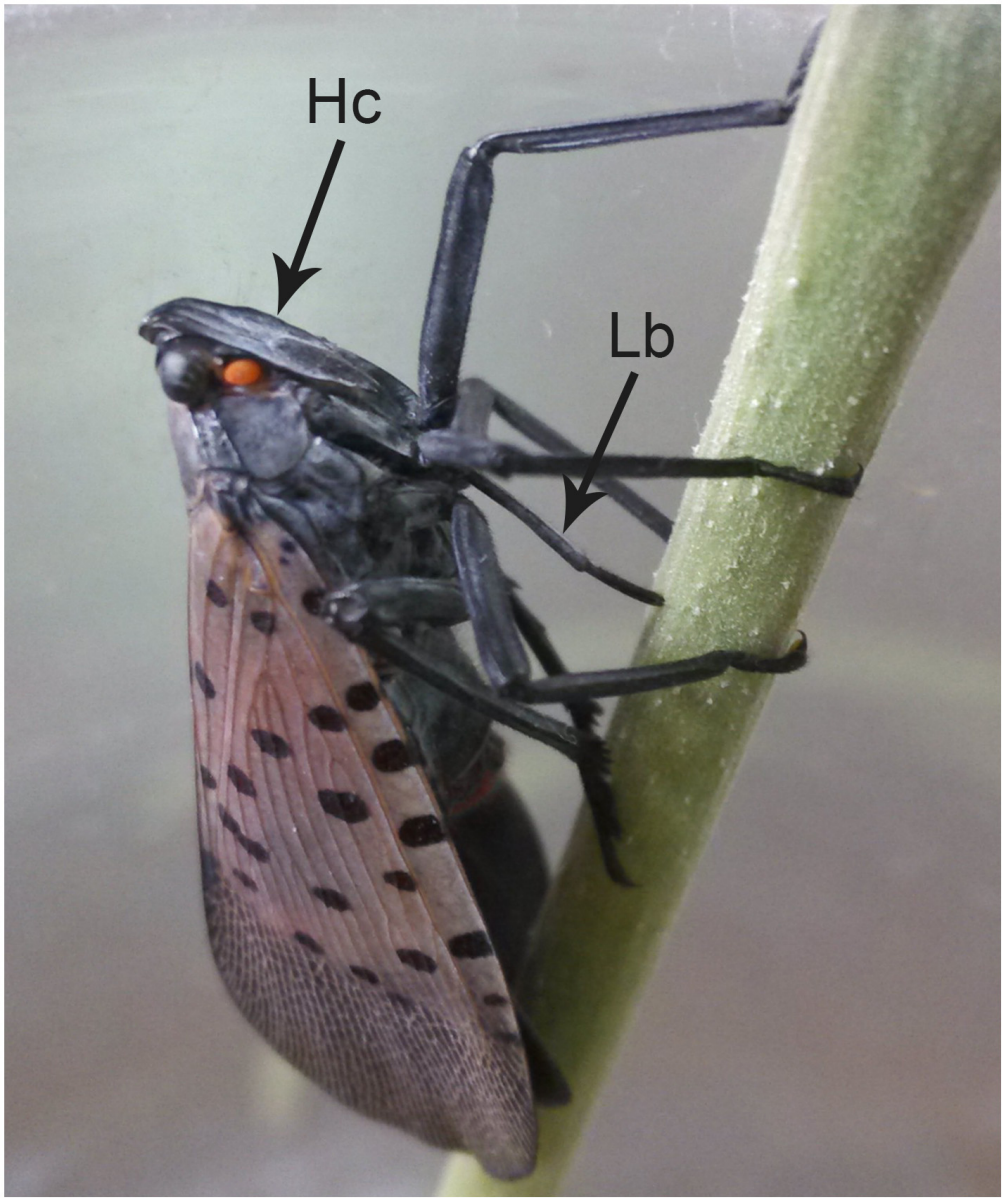
Adult female *Lycorma delicatula* showing mouthpart orientation during feeding. Hc, head capsule; Cl, clypeus.

**Fig 2:**
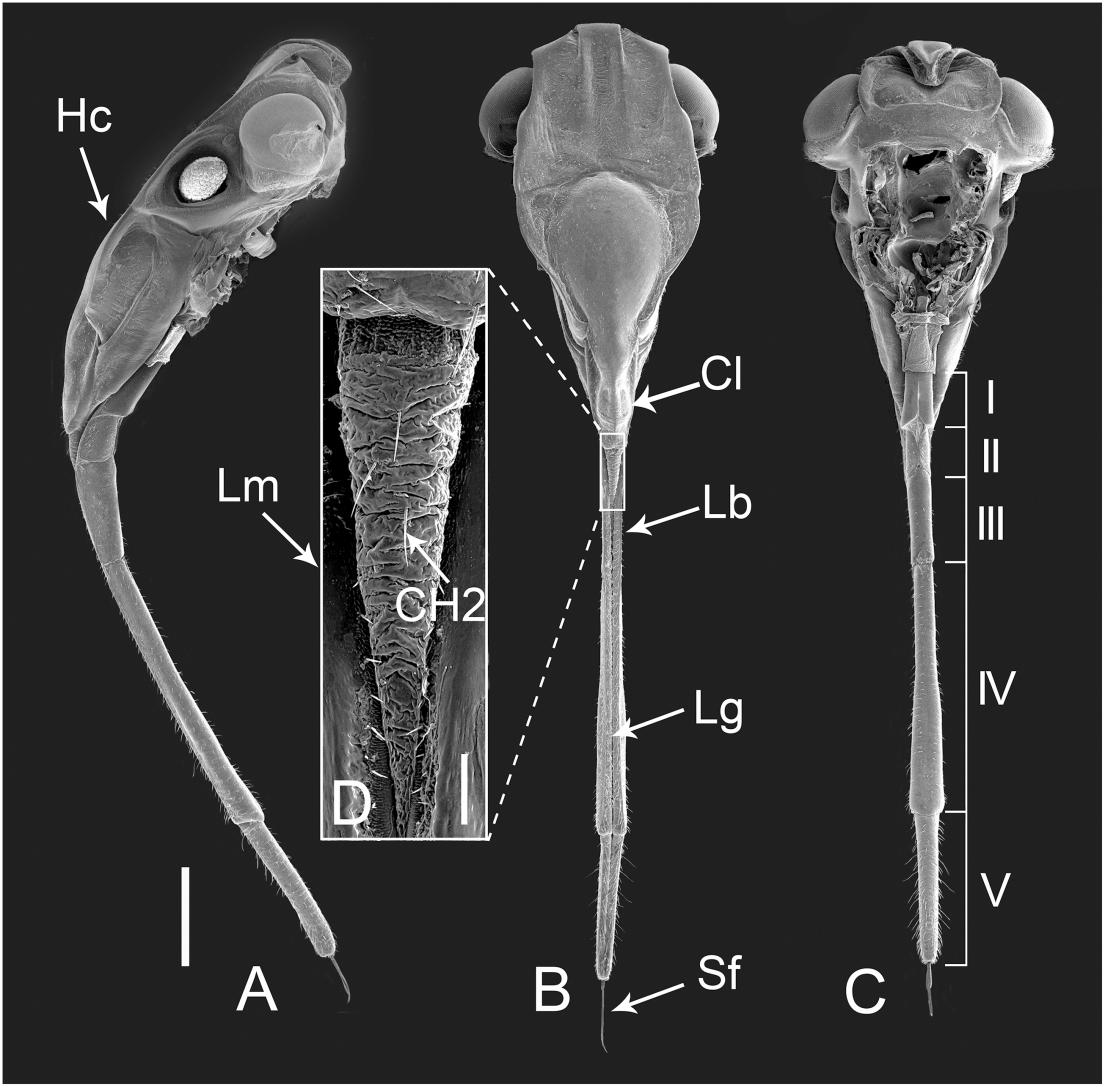
Scanning electron micrographs (SEMs) of the head of female *Lycorma delicatula*. (A) Lateral view. (B) Anterior view. (C) Ventral view showing the five labial segments. (D) Enlarged anterior view of labrum. Hc, head capsule; Cl, clypeus; Lm, labrum; Lb, labium; Lg, labial groove; Sf, stylet fascicle; CH2, sensilla chaetica II; I-V, segments of labium. Bars: (A), (B), and (C) = 1500 μm; (D) = 120 μm.

As in other members of Auchenorrhyncha, the mouthparts of *Lycorma delicatula* arise from the posteroventral part of the head capsule (Hc) (Figs 1 and 2A) and consist of a small cone-shaped labrum (Lm) (Fig 2D) and a tubular labium (Lb) (Fig 2B) with a deep longitudinal labial groove (Lg) on the dorsal surface that houses the stylet fascicle (Sf) (Fig 2B). The latter consists of two inner maxillary stylets (Mx) partially surrounded by two somewhat shorter mandibular stylets (Md). Various types of sensilla are symmetrically distributed on either side of the labial groove or positioned on the distal end of the labium (Fig 2A–2C). No obvious differences were noted between the mouthpart structure of females and males except for the length, which appears to be correlated with overall body size. The total length in females is 9392.90 ± 230.54 μm (n = 8), and for males is 8132.02 ± 450.69 μm (n = 3). When at rest, the mouthparts extend backward beneath the body, while during feeding, the mouthparts are rotated forward and held almost perpendicular to the plant surface (Fig 1).

### Labrum

**Fig 3:**
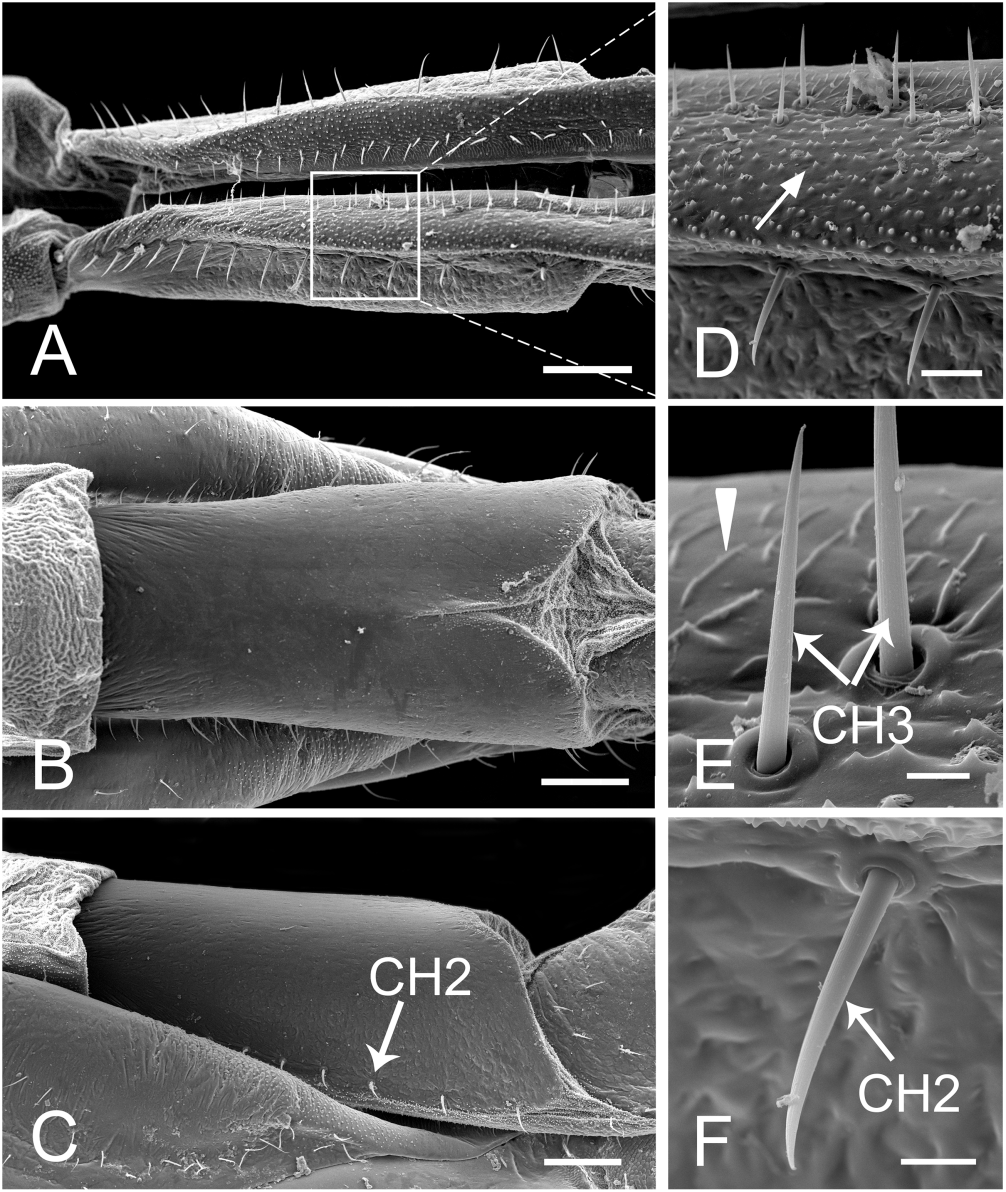
SEM of first segment of labium of female *Lycorma delicatula*. (A) Anterior view. (B) Ventral view. (C) Lateral view. (D) Enlarged view of outlined box of (A), showing the papillae (white arrow). (E) Enlarged view of sensilla chaetica III (CH3) and prominent transverse ridge (white triangle). (F) Enlarged view of sensilla chaetica II (CH2). Bars: (A), (B) and (C) = 150 μm; (D) = 30 μm; (E) = 6 μm; (F) = 12 μm.

**Fig 4:**
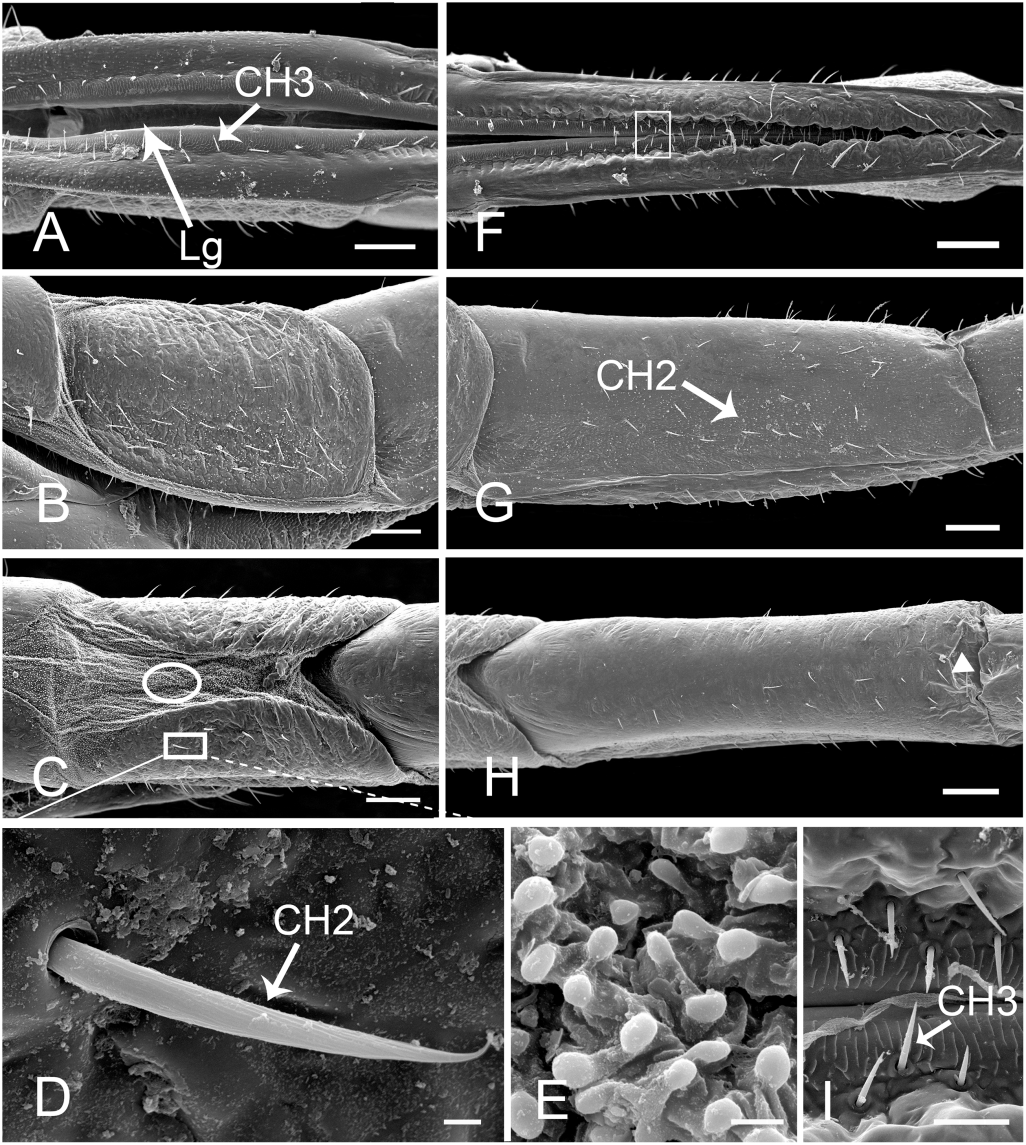
SEM of second and third segments of labium of female *Lycorma delicatula*. (A) Anterior view of second segment showing labial groove (Lg) and sensilla chaetica III (CH3). (B) Lateral view of second segment. (C) Ventral view of second segment. (D) Enlarged view of outlined box of (C) showing sensilla chaetica II (CH2). (E) Enlarged view of outlined circle of (C) showing membranous area with granular processes. (F) Anterior view of third segment. (G) Lateral view of third segment. (H) Ventral view of third segment, showing concave surface (white triangle) at junction of third and fourth segment. (I) Enlarged view of outlined box of (F) showing sensilla chaetica III (CH3) on both sides of labium groove. Bars: (A), (B) and (C) = 120 μm; (D) and (E) = 3 μm; (F), (G) and (H) = 150 μm; (I) = 30 μm.

**Fig 5:**
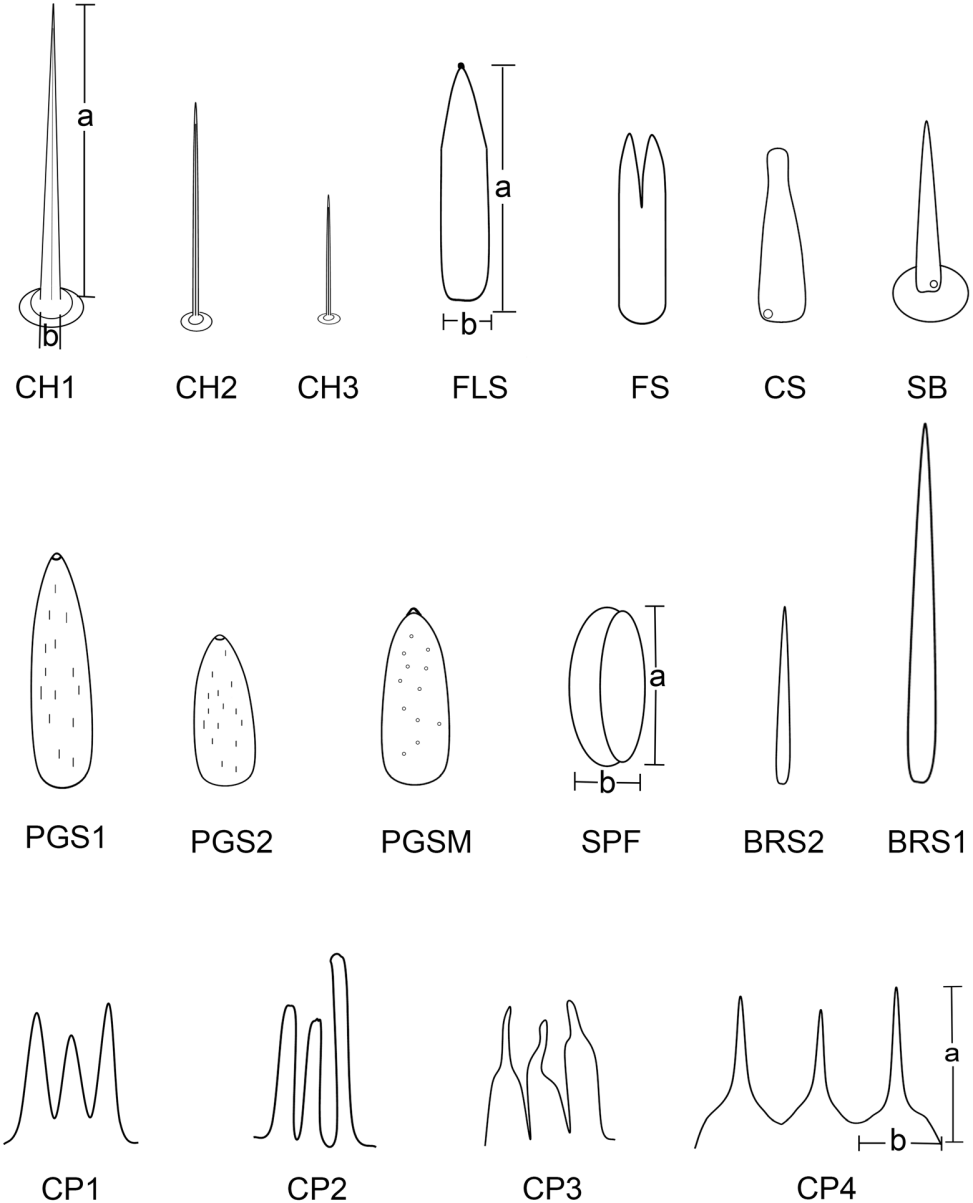
Diagrams of different types of sensilla and cuticular processes on mouthparts of female *Lycorma delicatula*. CH1, sensilla chaetica I; CH2, sensilla chaetica II; CH3, sensilla chaetica III; FLS, finger-like sensilla; FS, forficate sensilla; CS, clavate sensilla; SB, sensilla basiconica; PGS1, peg sensilla I; PGS2, peg sensilla II; PGSM, multiporous peg sensilla; SPF, placoid flattened sensilla; BRS1, bristle-like sensilla I; BRS2, bristle-like sensilla II; CP1, cuticular process I; CP2, cuticular process II; CP3, cuticular process III; CP4, cuticular process IV; a, length of sensilla; b, basal diameter of sensilla.

The labrum, generally conical, attaches to the anterior margin of the anteclypeus and overlays the labial groove (Lg) of the second and the third labial segments (Fig 2B, Table 1). The surface of the labrum is coarsely rugose with very few sensilla chaetica II (CH2) arranged randomly (Fig 2D, Table 2). These sensilla are slender and usually quite straight with the cuticular wall covered with longitudinal grooves, and insert into a slightly elevated socket (Figs 3C, 3F, 4D and 5).

**Table 1:** Measurements of labrum, labium and stylets (mean ± SE) obtained from scanning electron microscopy. N = sample size. Lm, labrum; Lb-sg1, first segment of labium; Lb-sg2, second segment of labium; Lb-sg3, third segment of labium; Lb-sg4, fourth segment of labium; Lb-sg5, fifth segment of labium; Md, mandibular stylet; Mx, maxillary stylet.

		Length (μm)	Width (μm)	Height (μm)	N
Male	Lb	8132.02±450.69			3
Female	Lm	917.82±28.35	237.73±7.11		10
	Lb-sg1	894.21±26.60	422.84±15.93	396.93±9.08	10
	Lb-sg2	749.77±21.93	396.62±13.20	470.58±13.13	10
	Lb-sg3	1495.50±32.74	348.17±13.16	490.73±22.09	10
	Lb-sg4	4065.84±81.78	388.47±21.48	370.04±11.23	10
	Lb-sg5	2260.12±53.88	256.48±21.82	305.51±8.50	10
	Md	9217.33±24.23	35.66±1.55		7
	Mx	10222.39±46.49	29.71±0.80		7

**Table 2:** Morphometric data for various sensilla of female adult *Lycorma delicatula*. N = sample size; CH1, sensilla chaetica I; CH2, sensilla chaetica II; CH3, sensilla chaetica III; SB, sensilla basiconica; SPF, placoid flattened sensilla; CS, clavate sensilla; FS, forficate sensilla; FLS, finger-like sensilla; PGSM, multiporous peg sensilla; PGS1, peg sensilla I; PGS2, peg sensilla II; BRS1, bristle-like sensilla I; BRS2, bristle-like sensilla II.

	Distribution	Length (μm)	Basal Diameter (μm)	N
CH1 (long)	Lb-sg4, sg5	211.04±12.70	7.05±0.51	7
CH2 (middle)	Lm, Lb	96.06±4.51	6.13±0.38	14
CH3 (short)	Lg	21.81±2.96	2.66±0.12	10
SB	Lb-sg5	18.27±2.28	4.72±0.42	4
SPF	Lb-sg5	51.46±1.78	20.92±1.09	10
CS	SD	18.56±3.57	3.84±0.47	6
FS	SD	9.32±0.27	2.63±0.07	5
FLS	SD	10.90±0.92	2.22±0.20	7
PGSM	SD	8.33±0.73	4.02±0.12	3
PGS1 (long)	SD	8.84±0.26	4.30±0.29	4
PGS2 (short)	SD	7.03±0.29	4.23±0.20	3
BRS1 (long)	SD, SV	45.38±2.07	4.10±0.29	4
BRS2 (short)	SD, SV	32.20±0.72	4.41±0.44	3

### Labium

The modified labium is a tube-like structure subdivided into five segments (Fig 2C). The length, width and height vary among the different segments (Table 1). The dorsal surface of the labium is bisected by a deep labial groove (Lg) extending for its entire length (Fig 2B). The maxillary and mandibular stylets lie in this groove, with their apices emerging from the apical central opening. The entire surface of the labium is covered with prominent sensilla, all angled toward the labial tip, but differing in their lengths and forms (Table 2).

The first (basal) segment is not very long and is always covered by the clypeus on the anterior side (Fig 2A and 2B, Table 1). When the labium is removed from the head, the anterior (morphologically dorsal) side can be clearly observed. On each side of the labial groove, two rows of sensilla chaetica III (CH3) (Table 2) are arranged side by side (Fig 3A and 3D). These sensilla resemble sensilla chaetica II (CH2) in their morphology but they are relatively shorter and straighter, with a smooth surface, and inserted into a pit with a sunken socket (Figs 3E and 5). Medially to these sensilla, the labial surface has prominent transverse ridges (Fig 3D and 3E) and laterally to them the surface is covered with small spines (Fig 3D). On each side of the groove, a single row of CH2 is regularly distributed along the edge (Fig 3C and 3F). The posterior (ventral) surface is relatively smooth with shallow indentations but sensilla are apparently lacking. The end of this segment is divided into two lobes by a membranous area continuous across the joint with the second segment (Fig 3B).

The second segment is as short as the first one and is approximately the same width throughout its length (Table 1). There are additional CH2 and CH3 distributed on the dorsal and lateral sides (Fig 4A, 4B and 4D). The well sclerotized lateral surfaces are connected with each other by a membranous area on the ventral side (Fig 4C) which is densely covered with small granular cuticular processes (Fig 4E).

The third segment is longer than the first two segments (Table 1), and possesses sensilla similar in structure and position to those on the second segment (Fig 4F and 4I). The labial groove narrows toward the apex of this segment as the result of convergence of the two sides. On the ventral side, the cuticle invaginates at the junction of the third and fourth segment (Fig 4H).

**Fig 6:**
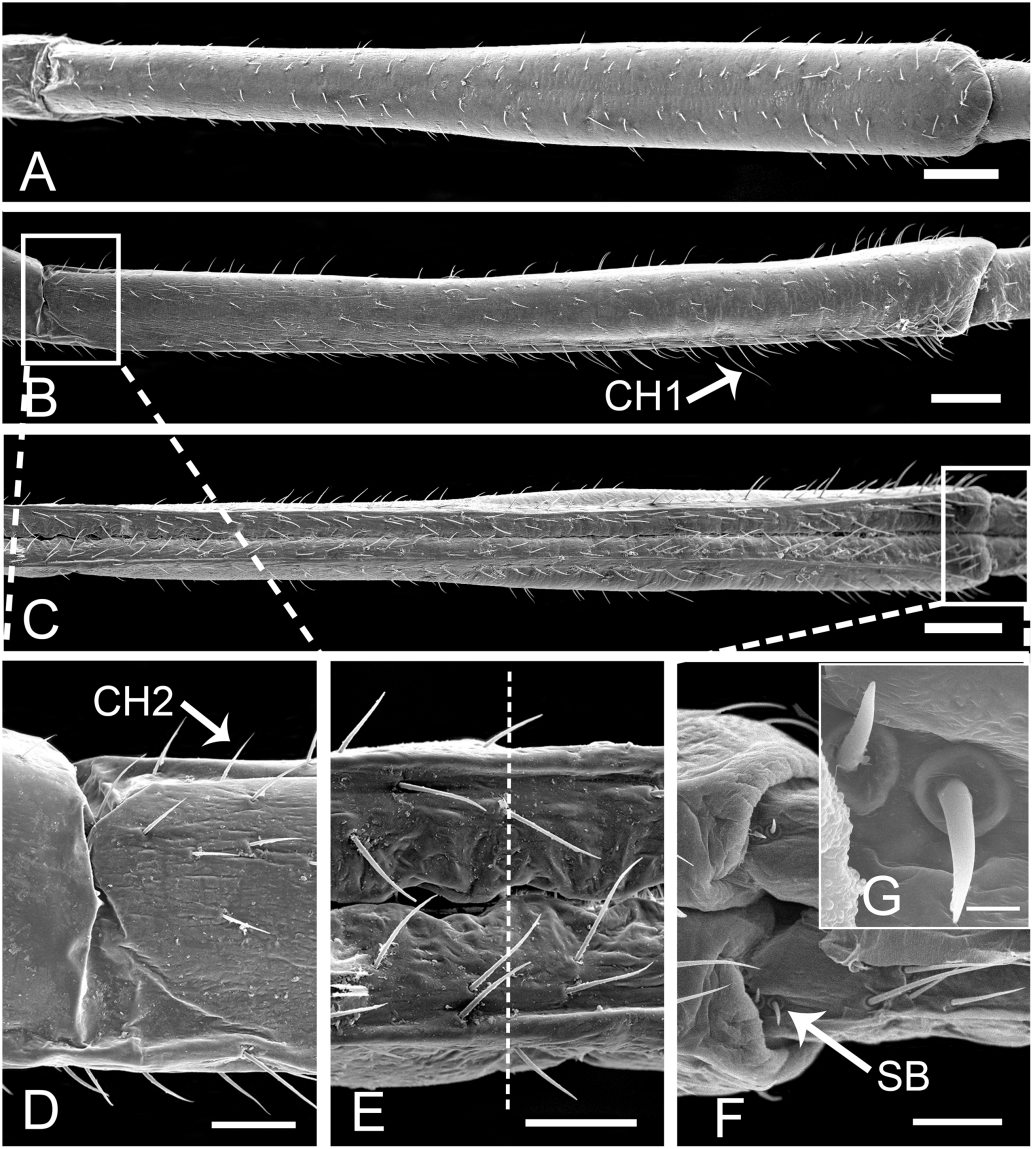
SEM of fourth and base of fifth segment of labium of female *Lycorma delicatula*. (A) Ventral view of fourth segment. (B) Lateral view. (C) Anterior view. (D) Enlarged view of outlined box of (B) showing lateral aspect of internode of third and fourth segment. (E) The anterior view of internode (white dotted line) of third and fourth segment. (F) Enlarged view of outlined box of (C) showing junction of fourth and fifth segment and SB at base of fifth segment. (G) Enlarged view of SB in (F). CH1, sensilla chaetica I; CH2, sensilla chaetica II; SB, sensilla basiconica. Bars: (A), (B) and (C) = 300 μm; (D), (E) and (F) = 90 μm; (G) = 9 μm.

The fourth segment, which is the longest one (Table 1), is thin at the base and is gradually broadened toward the apex (Fig 6A and 6C). More sensilla are found at the end than at the base (Fig 6B and 6C) and a few sensilla chaetica I (CH1) are sporadically distributed on this segment (Fig 6B). The CH1 are relatively long, frequently curved (Fig 5) and located only on the last two segments. In dorsal aspect, the internode between the third and fourth segments is poorly delimited (Fig 6E).

**Fig 7:**
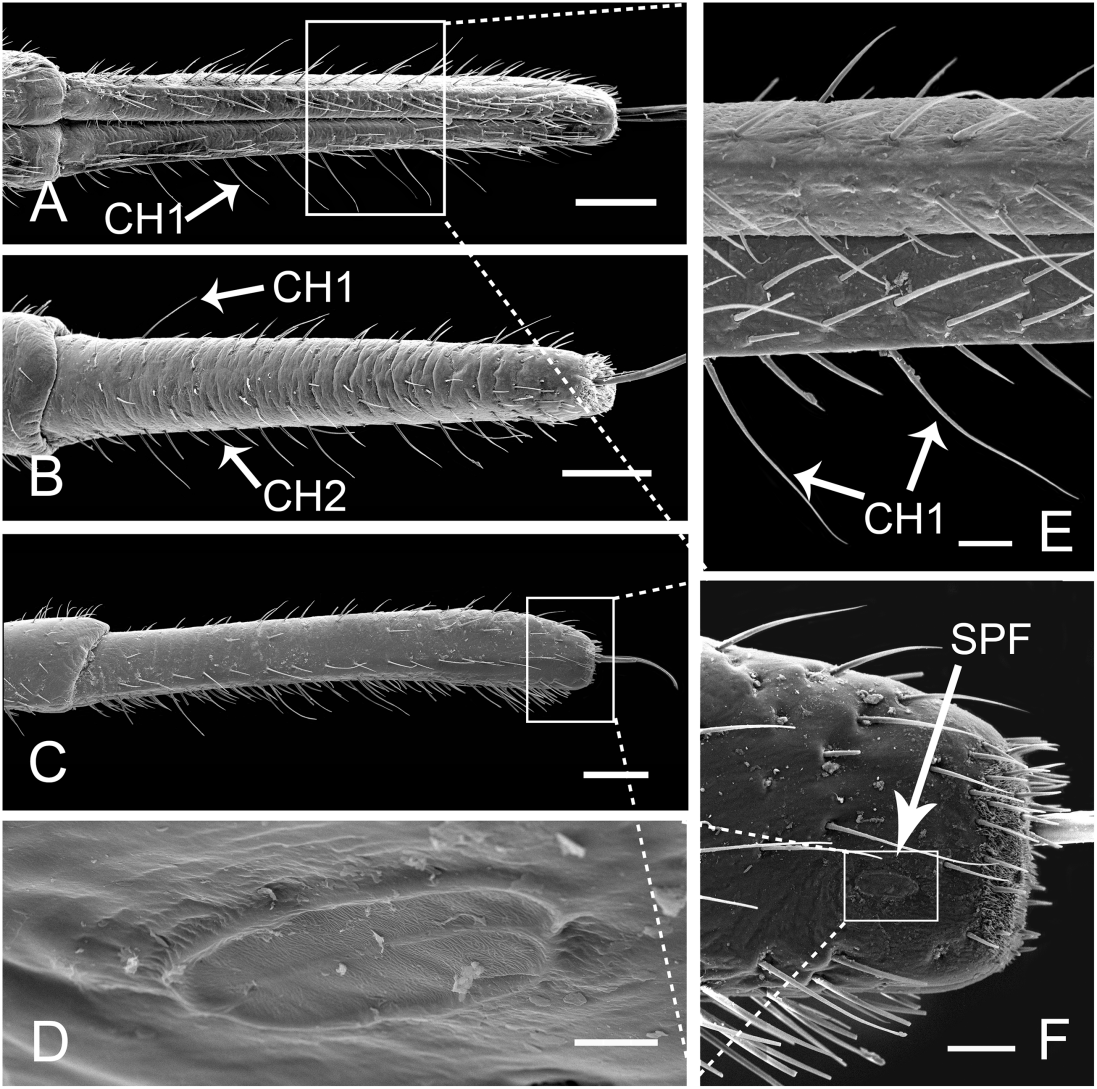
SEM of fifth segment of labium of female *Lycorma delicatula*. (A) Anterior view of fifth segment, showing arrangement of sensilla. (B) Ventral view of fifth segment, showing sensilla chaetica I (CH1) and sensilla chaetica II (CH2). (C) Lateral view of fifth segment. (D) Enlarged view of outlined box of (F) showing special placoid flattened sensilla (SPF). (E) Enlarged view of outlined box of (A) showing sensilla chaetica I (CH1). (F) Enlarged view of labial tip showing special placoid flattened sensilla (SPF). Bars: (A), (B) and (C) = 300 μm; (D) = 9 μm; (E) and (F) = 60 μm.

The fifth segment is cylindrical, with a thick base and slightly thinner end (Fig 7A and 7B). At the junction with the fourth segment, there are two pairs of sensilla basiconica (SB) (Fig 6F and 6G) located on both sides of the labial groove. They are quite straight, with smooth surfaces, projecting out from a convex round base and almost perpendicular to the surface. CH1 and CH2 are interlaced on the dorsal and lateral surfaces (Fig 7A, 7B and 7E). Two special placoid flattened sensilla (SPF) are located laterally near the apex, 44.44 ± 4.07 μm (n = 10) from the tip (Fig 7F); they are elliptical, slightly concave, and parallel to the longitudinal axis of the labium, surrounded by a double furrow (Figs 5 and 7D). The surface of the sensillum is irregularly rugulose, and a small pore can be found on lateral side of the midline (Figs 5 and 7D).

**Fig 8:**
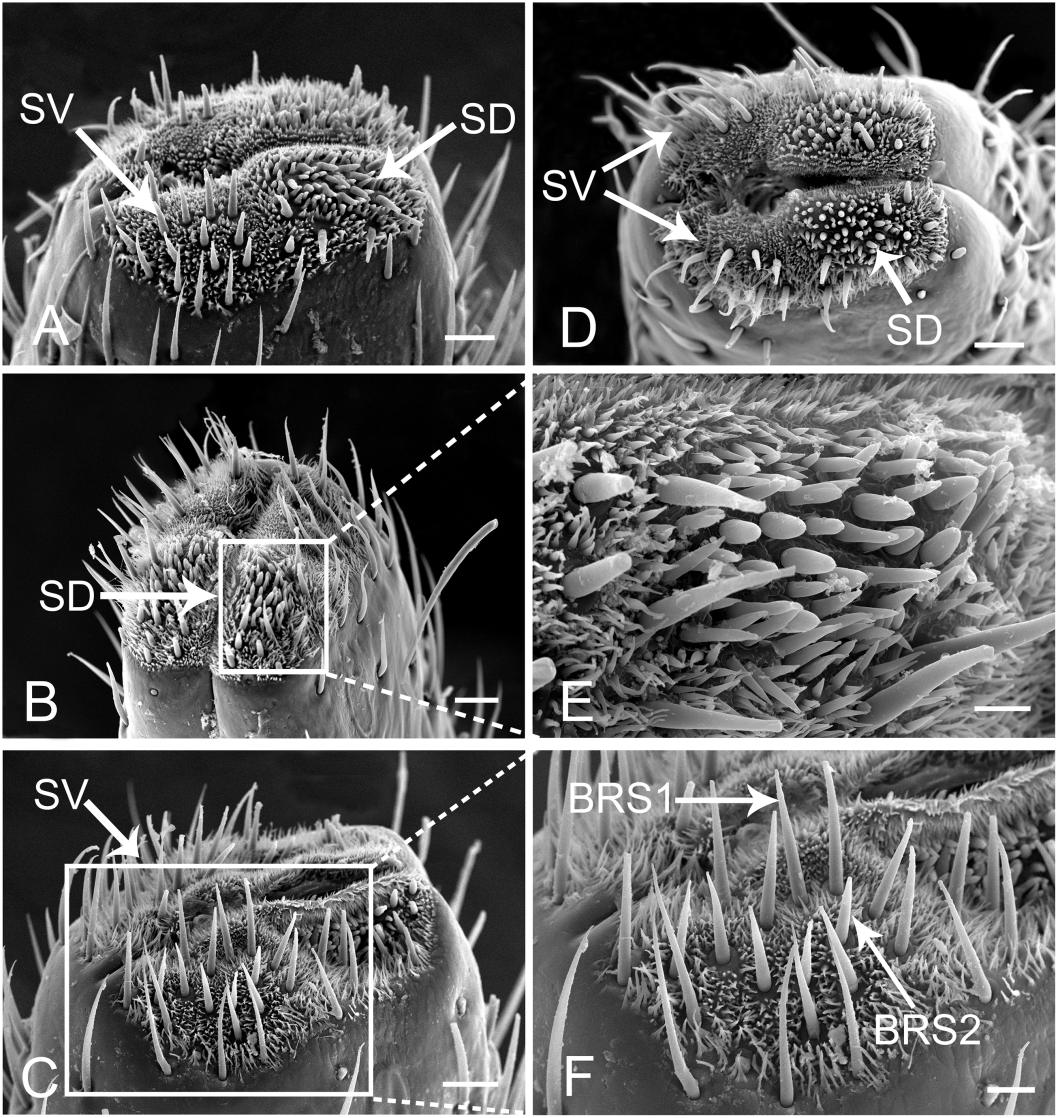
Distribution of various sensilla on tip of labium of female *Lycorma delicatula*. (A) (B) (C) (D) SEM views of labial tip of different individuals showing variation in numbers of sensilla. (E) Enlarged view of the outlined box of (B) showing the dorsal sensory field (SD). (F) Enlarged view of the ventral sensory field (SV) showing the bristle-like sensilla I (BRS1) and bristle-like sensilla II (BRS2). Bars: (A), (B), (C) and (D) = 30 μm; (E) = 9 μm; (F) = 15 μm.

**Fig 9:**
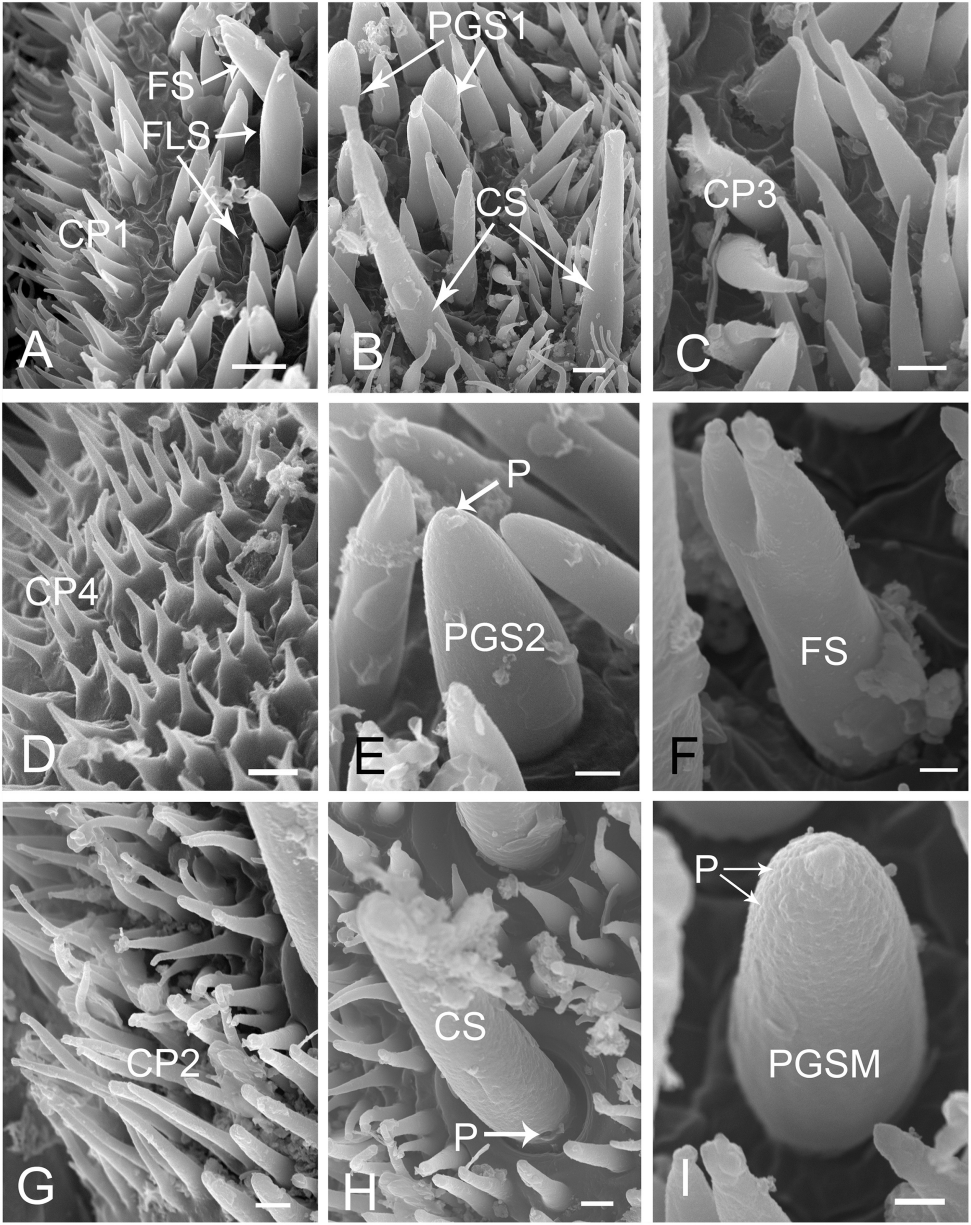
Enlarged views of different types of sensilla and cuticular processes on mouthparts of female *Lycorma delicatula*. CP1, cuticular process I; CP2, cuticular process II; CP3, cuticular process III; CP4, cuticular process IV; FS, forficate sensilla; FLS, finger-like sensilla; CS, clavate sensilla; PGS1, peg sensilla I; PGS2, peg sensilla II; PGSM, multiporous peg sensilla; P, pore. Bars: (A) and (B) = 3 μm; (C) = 1.5 μm; (D) = 2 μm; (E) = 1.5 μm; (F) = 0.8 μm; (G) and (H) = 1.5 μm; (I) = 1 μm.

The tip of the labium is divided into two lobes by the labial groove and has two pairs of well defined sensory fields. Each of these fields comprises numerous sensilla and cuticular processes of various forms (Fig 8A–8D). The sensory fields consist of a pair of dorsal sensory fields (SD) (Fig 8E) and a pair of ventral sensory fields (SV) (Fig 8F). Sensilla are more numerous on the dorsal field compared to ventral fields, and their morphology and quantity may vary in different individuals (Fig 8A–8D). In each sensory field, different sensilla are surrounded by different types of cuticular processes. The dorsal field includes three kinds of cuticular processes and six types of sensilla. Cuticular processes I (CP1) (3.97 ± 0.31 μm long, n = 10) are denticles [41] with a wide base (1.32 ± 0.08 μm wide, n = 10) and a sharply pointed tip; generally, few bases are linked together (Figs 5 and 9A). These denticles are mainly found on both sides of the middle line and around the dorsal sensory fields. Cuticular processes II (CP2) (9.71 ± 0.62 μm long, n = 10, and 1.25 ± 0.10 μm wide at base, n = 10) are arranged at the periphery of the dorsal area. They are more slender, only slightly tapered from based to apex, and blunt tipped or slightly clavate (Figs 5 and 9G). Cuticular processes III (CP3) (4.56 ± 0.57 μm long, n = 10, and 1.39 ± 0.06 μm wide at base, n = 10) are found on the edge adjacent to the dorsal side of the labial tip (Figs 5 and 9C). They have a quite wide base but suddenly taper near the midlength to a slender distal portion which, in most cases, is curved with blunt tips. The ventral sensory fields possess the fourth type of cuticlar processes (CP4) covering most of the surface (Fig 9D). They are quite short (2.11 ± 0.12 μm long, n = 10), possess a wide and flat base (1.29 ± 0.12 μm wide, n = 10) and narrow to a blunt tip (Figs 5 and 9D).

### Labial Tip Sensilla

Six different morphological types of sensilla are found on the labial tip.

#### Bristle-like sensilla (BRS)

Bristle-like sensilla are nonporous, tapered, with a sharp tip and a smooth cuticular wall. They are found in both dorsal and ventral sensory fields and are subdivided into two groups according to their size, BRS1 (40–50 μm) and BRS2 (30–40 μm) (Figs 5 and 8F) (Table 2).

#### Multiporous peg sensilla (PGSM)

They are ovoid shaped with a small prominence at the round tip, multiporous (Figs 5 and 9I), about 7 to 9 μm in length (Table 2). Their cuticle wall is accidented and covered with tiny pores (Figs 5 and 9I). This type of sensillum was observed on the center of dorsal sensory fields.

#### Peg sensilla (PGS)

They are uniporous, cone shaped with a rounded tip, and are only found in the dorsal sensory field. Longitudinal veins were found on their surface. They are subdivided into two groups according to their size, PGS1 (8–9 μm) (Figs 5 and 9B) and PGS2 (6–8 μm) (Figs 5 and 9E) (Table 2).

#### Forficate sensilla (FS)

They are 8–11 μm long and cylindrical at the base. The tip is divided into two or three processes. They are nonporous, with a smooth cuticular wall, and only found in the dorsal sensory field (Figs 5, 9A and 9F).

#### Finger-like sensilla (FLS)

Ranging from 6–13 μm long, these sensilla are slender and nonporous, with a smooth cuticular wall. The upper third tapers and sometimes has a small bump on the tip (Figs 5 and 9A). They are only found in the dorsal sensory field.

#### Clavate sensilla (CS)

These sensilla are also slender, but their length varies considerably (6–28 μm). They have a rough cuticular wall, thick base and are slightly curved, flat at the top (Figs 5 and 9B), and possess a pore at their base (Fig 9H). They are only found in the dorsal sensory field.

### Stylet Fascicle

**Fig 10:**
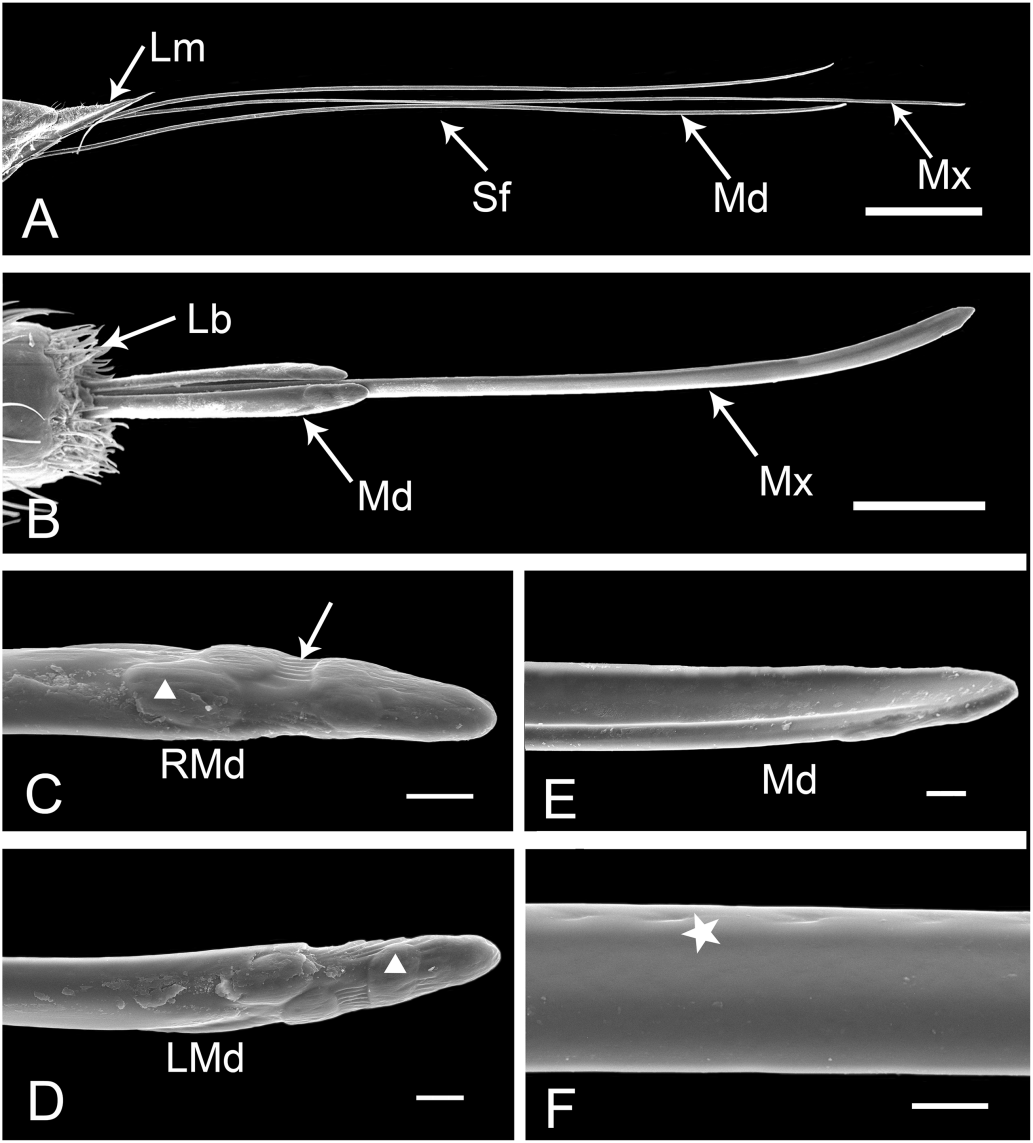
SEM of the stylet fascicle and mandibular stylets of female *Lycorma delicatula*. (A) Labrum (Lm) and stylet fascicle (Sf) showing longer maxillary stylets (Mx) and shorter mandibular stylets (Md). (B) Apex of stylet fascicle extended from labial (Lb) tip showing outer (shorter) mandibular stylets (Md) and longer maxillary stylets (Mx). (C) Tip of right mandibular stylet (RMd), showing oval plate-like prominences (white triangle) and longitudinal striations (white arrow) on convex external surface. (D) Tip of left mandibular stylet (LMd) showing prominences (white triangle). (E) Smooth hollow inner surface of mandibular stylet (Md). (F) Smoothed-out surface of mandibular stylet and series of pits (white pentastar). Bars: (A) = 1200 μm; (B) = 150 μm; (C), (D), (E) and (F) = 15 μm.

**Fig 11:**
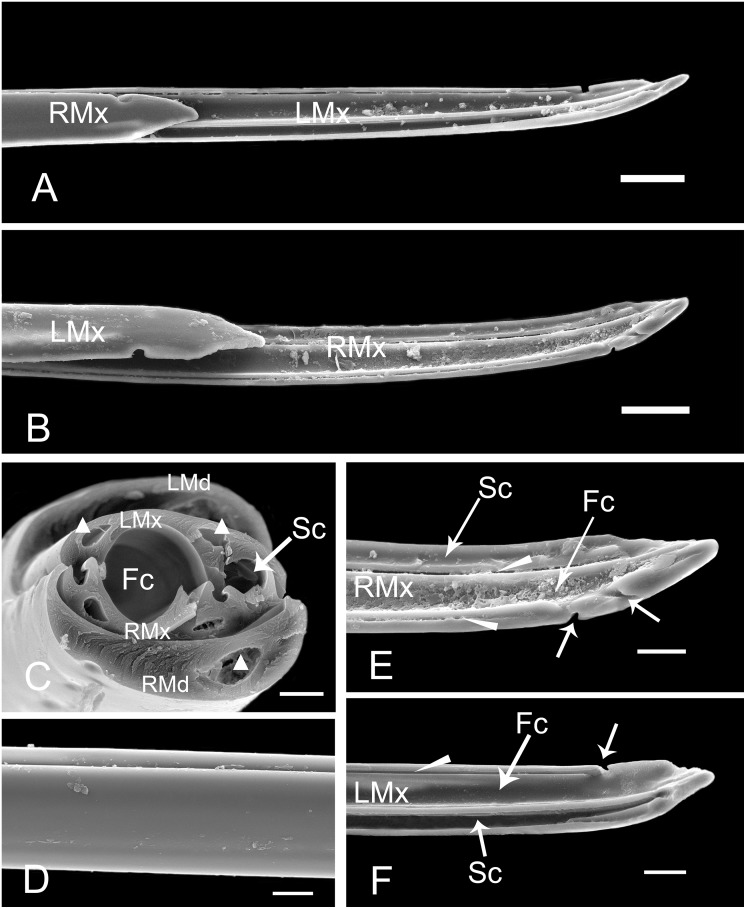
SEM of maxillary stylets of female *Lycorma delicatula*. (A) Apex of interlocked maxillary stylets showing outer surface of right maxillary stylet (RMx) and inner surface of left maxillary stylet (LMx). (B) Apex of interlocked maxillary stylets showing outer surface of left maxillary stylet (LMx) and inner surface of right maxillary stylet (RMx). (C) Cross section of the stylet fascicle through the fourth labial segment, showing the shape of mandibular stylets (Md), maxillary stylets (Mx), food canal (Fc), salivary canal (Sc) and dendritic canals (white triangle). (D) Smooth external surface of maxillary stylet. (E) Inner surface of right maxillary stylet (RMx) showing the food canal (Fc), salivary canal (Sc), two interlocking canals (white long triangle) and two slit-like openings (white arrow). (F) Inner surface of left maxillary stylet (LMx) showing the food canal (Fc), salivary canal (Sc), interlocking canal (white long triangle) and semicircular breach (white arrow). Bars: (A) and (B) = 30 μm; (C) = 6 μm; (D) = 9 μm; (E) = 12 μm; (F) = 15 μm.

The stylet fascicle (Sf) is an elongate structure composed of paired mandibular (Md) and interlocking maxillary (Mx) stylets (Fig 10A and 10B); it often protrudes slightly from the labial (Lb) tip when the labium is in rest position (Fig 10B). The maxillary stylets are much longer than the mandibular stylets (Table 1). The mandibular stylets are crescent-shaped and axially symmetrical in cross-section (Fig 11C), being convex externally and concave internally to form a sheath enclosing the maxillary stylets (Fig 10E). Each mandibular stylet bears a large dendritic canal (9.62 ± 1.23 μm long, n = 6, and 4.16 ± 0.62 μm wide, n = 6) on the thicker side (Fig 11C). On the lateral surface near the thinner side, a series of pits (radius 3.04 ± 0.34 μm, n = 3) extend throughout the length (Figs 10F and 11C), while the inner surface is smooth (Fig 10E). On the outer surface of the terminal part of mandibular stylets, three oval plate-like prominences are present on each stylet (Fig 10C and 10D). The distal one is broadest from left to right; the middle one is smaller, more evenly rounded and situated more medially; and the basal is largest and oriented more longitudinally. Longitudinal striations occur along both sides of the tip (Fig 10C and 10D).

**Fig 12:**
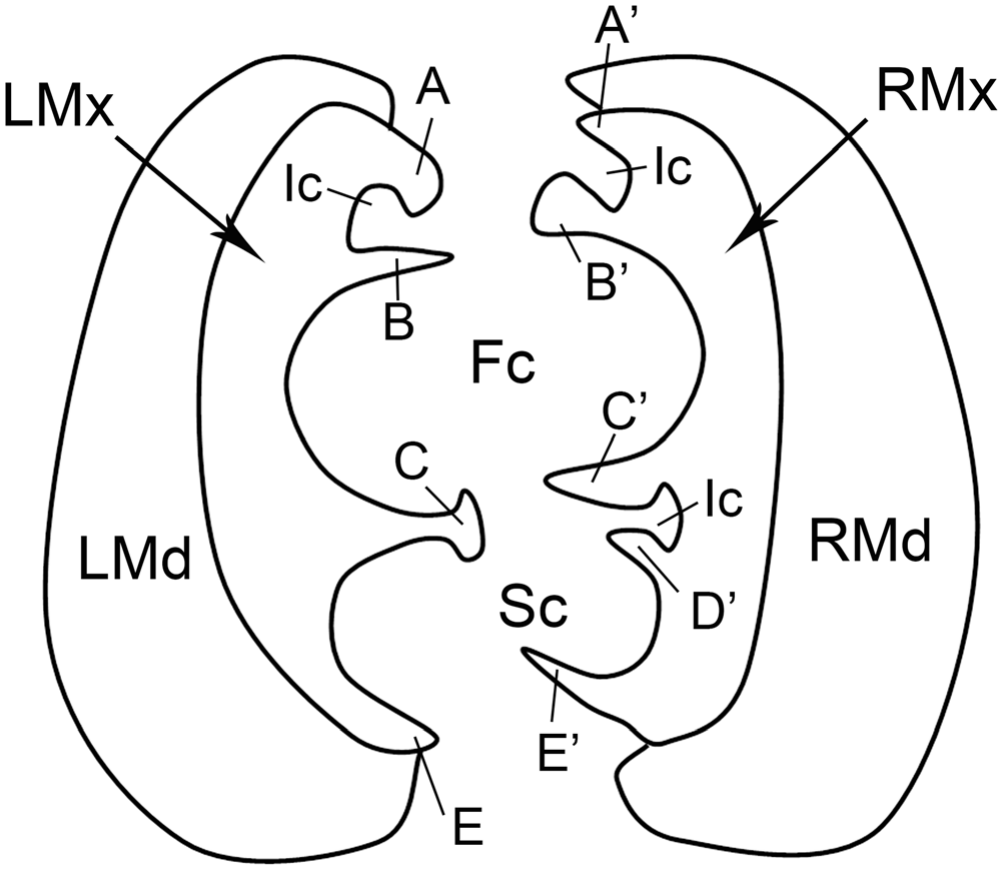
Diagram of cross-section of stylet fascicle through fourth labial segment of female *Lycorma delicatula*. LMd, left mandiblular stylet; RMd, right mandiblular stylet; LMx, left maxillary stylet; RMx right maxillary stylet; Fc, food canal; Sc, salivary canal; Ic, interlocking canal; A, hooked upper left process of dorsal lock; A’, straight upper right process of dorsal lock; B, straight lower left process of dorsal lock; B’, hooked lower right process of dorsal lock; C, T-shaped left process of middle lock; C’, hooked upper right process of middle lock, D’, hooked lower right process of middle lock; E, straight lower left process of ventral lock; E’, hooked lower right process of ventral lock.

The maxillary stylets (Mx) are semicircular in cross-section and are interlocked by a connecting system composed of T-shaped, hooked, and straight processes (Figs 11C and 12). However, the number of these processes differs between right and left stylets (Fig 12). The five processes on the right maxilla form four grooves: the food canal, salivary canal and two additional interlocking canals (Figs 11E and 12). On the dorsal side of the right maxilla, there are two pre-apical slit-like openings near the tip (Fig 11E). On the left maxilla, three grooves are formed by four processes (Figs 11F and 12), and a semicircular breach is located on the dorsal side, about 36.31 μm from the tip (Fig 11F). The oval food canal, through which plant sap is sucked into the esophagus, is 13.56 ± 0.72 μm (n = 5) in diameter and is located centrally in the subdorsal portion. The salivary canal that directs saliva into the plant is formed on the ventral side and is 4.88 ± 0.24 μm (n = 4) in diameter. Within each maxillary stylet there are two comma-shaped dendritic canals (4.08 ± 0.35 μm long, n = 11, 1.92 ± 0.14 μm wide, n = 11), smaller than those of the mandibular stylets. The outer surface of maxillary stylets is quite smooth throughout its length (Fig 11D).

## Discussion

Hemipterans have highly modified piercing-sucking mouthparts and various types of sensilla that play important roles in finding hosts [42], feeding and transmitting plant pathogens [43]. The morphology of these structures varies considerably among families, genera and species. Such variation can be used for classification and identification, as well as for ecological or physiological study [44]. While numerous observations on the mouthparts of other Auchenorrhyncha have been published [1,4,9,10,13,24,27], no previous research has been conducted on the spotted lanternfly’s mouthparts using scanning electron microscopy. Our study revealed the presence of various sensilla and details of their arrangement on the labial tip in this species for the first time. This will lay a foundation for future studies of the evolution of mouthpart morphology [19] and mechanisms for feeding [45].

As noted by Emeljanov [46] the number of labial segments in Hemiptera varies from 1–5 with the usual number being 3 or 4. Reductions in segment number have occurred in Sternorrhyncha (Coccoidea) and Heteroptera. Only *Lycorma delicatula* and aphids (Aphidoidea) have been reported to have five labial segments but, in the latter, interpretation of the distal sensory tubercle as a separate segment is controversial [46]. In Fulgoromorpha, as in other hemipterans, the original number was presumably 3 with the first segment comparatively short and the other two much longer and subequal in length. Fulgoroids with a 4-segmented labium have the first primary segment divided and those with 5 segments have both the first and the second primary segments divided into two segments [46]. The spotted lanternfly appears to be unusual in having a labium that is divided into four segments in nymphs and five segments in adults as a result of the subsegmentation of the longest (penultimate) segment of nymphs. This five-segmented labium was first noted by Lieu [26] and has not been reported in other fulgoromorphan insects in the literature, but appears also to occur in at least some other Asian genera of Fulgoridae (e.g., *Penthicodes pulchella* Guerin-Meneville and *Pyrops candelaria* (L.); unpublished observations). Further comparative study of labial segmentation in Fulgoridae is, therefore, needed. Given the unusually extensive elongation of the labium of the spotted lanternfly compared to that of most other planthoppers, selection for greater flexibility during feeding may have led to the increase in segment number through subsegmentation of the penultimate labial segment which, in other Auchenorrhyncha, bears several muscle attachment points internally [47].

Sensory organs on the mouthparts of the spotted lanternfly consist of mechanoreceptor sensilla chaetica and two specialized structures of the labium: the paired subapical sensory organs and the apical sensory fields. They play important roles in the feeding process [2] by discriminating between appropriate and non-appropriate plant tissues and guiding the stylets to the phloem [48]. The most abundant sensilla on the labium of the spotted lanternfly are sensilla chaetica, which have no pores and are therefore considered to be mechanoreceptive [1,40,49]. All three types of sensilla chaetica reported here have been observed in Derbidae and Flatidae, and one or two of them can be found in other families of Fulgoromorpha [1]. The longest sensilla are only located on the last two labial segments, which are the first to contact the plant surface and thus may play a role in mechanically sensing the feeding sites. The two pairs of sensilla basiconica at the joint of the fourth and fifth segment are commonly present in Fulgoroidea [1], and their location suggests they are proprioceptors that detect the degree of flexion of the joint, thereby allowing monitoring of their relative positions [44].

The presence of a paired subapical sensory organ was first reported in delphacid planthoppers by Sogawa [21] and has been found in most Fulgoromorpha families by Cobben [50], Liang [23] and Brożek and Bourgoin [1]. These placoid sensilla are all multiporous, but their shape and number vary in different species and they are absent in Cicadomorpha [50]. The ones in *Lycorma delicatula* are similar to those found in the Dictyopharidae and Ricaniidae, and are presumed to have olfactory and thermoreceptive functions [1]. The absence of these subapical sensory organs in some species could be linked to differences in host-plant preference [50]. Further investigations using TEM will be necessary to examine the ultrastructure of placoid sensilla and determine their specific functions.

Based on the morphological classification systems of Altner and Prillinger [40] and Brożek [1], nine types of sensilla were found on the labium of the spotted lanternfly. A variety of sensilla at the tip of the labium has been found in other planthopper families, but not as wide a variety as found here. The sensory structures on the apical segment of the labium have been previously described in representatives of fifteen fulgoromorphan families, including Fulgoridae, and the types and distributions of sensilla are more or less different among these groups. According to previous research on Fulgoromorpha [1], six short and three long uniporous peg sensilla, one long multiporous peg sensilla, six short and four long uniporous bristle-like sensilla are usually found in the dorsal sensory field. Four short and eleven long uniporous bristle-like sensilla are usually present in the ventral field [1]. In the spotted lanternfly, the number of each sensilla type differs among individuals but CS, FS, and FLS are always present. As the first sensory organs to contact plants, sensilla on the tip of the labium play essential roles in host plant identification. The nonporous bristle-like sensilla, forficate sensilla and finger-like sensilla are presumably either mechanoreceptive or contact-chemoreceptive structures. Uniporous clavate sensilla and peg sensilla are most likely gustatory sensilla. The multiporous peg sensilla likely have an olfactory function. As indicated by previous studies [1,5,24,40,51], assignment of sensilla to functional groups is generally possible based on their position and outer cuticular structures such as the presence of pores, but differences in shape are not always in accord with the differences in functionally relevant internal structures [40]. The accurate definition of sensilla types needs more investigation incorporating study of ultrastructure by transmission electron microscopy.

The sharp end and the protrusions on the apical surface of the mandibular stylets of hemipterans have been linked to the stabilization of the maxillary stylets during probing [52], thus providing a fulcrum for the movement of the maxillae [3,53,54] and also assist in the course of ecdysis [45]. The number and size of protrusions varies among different species of hemipterans [27,38], and may reflect variation in physical properties (e.g., density) of host plant tissue [55]. The longitudinal striations on the apical surface of mandibular stylets may prevent the mandibular stylets from rotating during probing. The smooth inner surface of the mandibular stylets and the outer surface of the maxillary stylets facilitate retraction of the stylets following the alternating sliding of mandibular stylets during probing and feeding [56]. The interlocking mechanism of the maxillae and mandibles in Fulgoroidea has been studied in detail by Brożek, and three locking mechanisms have been identified that are formed by straight, hooked and T-shaped processes [16]. Our observations of cross sections through the tips of the maxillary stylets show that the same interlocking mechanisms exist in the spotted lanternfly. The dorsal lock can prevent the unhooking of the locks when the maxillae are moving, and the middle lock prevents the maxillae from opening [16]. The presence of three locks in the mouthparts has also been observed in both Heteroptera [18] and Sternorrhyncha [15], while there are only two locks in Cicadomorpha [9].

The position of the food canal is stable in various groups of Hemiptera [16–18,27,38,41,48]. The slit-like opening on the maxillary stylet observed in *Lycorma delicatula* has also been found in the seed bug, *Spilostethus pandurus* (Scopoli), and appears to play a role in uptake of phloem-sap or water, but the precise mechanism is not yet understood [3]. The number of dendritic canals varies extensively among different hemipteran species. Two dendritic canals in each maxilla and one in each mandible have also been found in Cixiidae [17], the leafhopper *Psammotettix striatus* [38] and the delphacid *Sogatella furcifera* [27]. But there is only one dendritic canal in each maxilla and each mandible of the leafhopper *Macrosteles fascifrons* Stål [22], true bugs [18] and the coleorrhynchan *Xenophyes cascus* [19]. In Sternorrhyncha, including *Psylla chinensis* [48], aphids [57] and *Bemisia tabaci* [58], only one dendritic canal was found in each mandibular stylet. The presence of dendritic canals may be related to the dual innervations [2] of the stylet fascicle and probably have a proprioceptive function [45].

This study reveals for the first time the diverse morphology of sensory structures on the labial tip in the spotted lanternfly. Additional transmission electron microscope and electrophysiological studies should be done to clarify the functions of various sensory organs.
